# The effects of a selective inhibitor of c-Fos/activator protein-1 on endotoxin-induced acute kidney injury in mice

**DOI:** 10.1186/1471-2369-13-153

**Published:** 2012-11-23

**Authors:** Hiroyuki Miyazaki, Jun Morishita, Masaaki Ueki, Kahoru Nishina, Shunichi Shiozawa, Nobuhiro Maekawa

**Affiliations:** 1 Division of Anesthesiology and Perioperative Medicine, Kobe University Graduate School of Medicine, Chuo-ku, Kobe, Hyogo, 650-0017, Japan; 2 Department of Medicine, Kyushu University Beppu Hospital, 4546 Tsurumihara, Beppu, Oita, 874-0838, Japan

**Keywords:** Sepsis, Acute kidney injury, TNF-α, Lipopolysaccharide, Activator protein-1

## Abstract

**Background:**

Sepsis has been identified as the most common cause of acute kidney injury (AKI) in intensive care units. Lipopolysaccharide (LPS) induces the production of several proinflammatory cytokines including tumor necrosis factor (TNF)-alpha, a major pathogenetic factor in septic AKI. c-Fos/activator protein (AP)-1 controls the expression of these cytokines by binding directly to AP-1 motifs in the cytokine promoter regions. T-5224 is a new drug developed by computer-aided drug design that selectively inhibits c-Fos/AP-1 binding to DNA. In this study, we tested whether T-5224 has a potential inhibitory effect against LPS-induced AKI, by suppressing the TNF-alpha inflammatory response and other downstream effectors.

**Methods:**

To test this hypothesis, male C57BL/6 mice at 7 weeks old were divided into three groups (control, LPS and T-5224 groups). Mice in the control group received saline intraperitoneally and polyvinylpyrrolidone solution orally. Mice in the LPS group were injected intraperitoneally with a 6 mg/kg dose of LPS and were given polyvinylpyrrolidone solution immediately after LPS injection. In the T-5224 group, mice were administered T-5224 orally at a dose of 300 mg/kg immediately after LPS injection. Serum concentrations of TNF-alpha, interleukin (IL)-1beta, IL-6 and IL-10 were measured by ELISA. Moreover, the expression of intercellular adhesion molecule (ICAM)-1 mRNA in kidney was examined by quantitative real-time RT-PCR. Finally, we evaluated renal histological changes.

**Results:**

LPS injection induced high serum levels of TNF-alpha, IL-1beta and IL-6. However, the administration of T-5224 inhibited the LPS-induced increase in these cytokine levels. The serum levels of IL-10 in the LPS group and T-5224 group were markedly elevated compared with the control group. T-5224 also inhibited LPS-induced ICAM-1 mRNA expression. Furthermore histological studies supported an anti-inflammatory role of T-5224.

**Conclusions:**

In endotoxin-induced AKI, T-5224 inhibited the production of TNF-alpha and other downstream effectors. In contrast, T-5224 did not inhibit IL-10, an anti-inflammatory cytokine. These data support that the use of T-5224 is a promising new treatment for septic kidney injury.

## Background

Recently, the incidence of acute kidney injury (AKI) has increased considerably. AKI occurs in 19–51% of patients with sepsis or septic shock, while sepsis is a contributing factor in approximately half of patients with severe AKI [1]. Although medical practice has advanced in the last decade, the pathophysiology of sepsis-induced AKI is incompletely understood, and the mortality rate remains high. Current treatments for sepsis-induced AKI including intensive insulin therapy and early goal-directed therapy have been reported to be beneficial; however, there are still no radical treatments to completely treat septic AKI [1].

Several inflammatory mediators have been assigned a key role in sepsis. In response to lipopolysaccharide (LPS), macrophages and other cells express and release multiple proinflammatory cytokines, such as tumor necrosis factor (TNF)-α [2]. Although these molecules are crucial for host defense against invading bacteria, excessive production of these mediators can result in a lethal systemic inflammatory response syndrome, which leads to shock, vascular dysfunction, disseminated intravascular coagulation and multiple organ dysfunction/injury [3]. Specifically, the role of TNF-α has been shown to be critical in the inflammatory response in endotoxemia-related AKI [3].

The responses to LPS are mostly dependent on the mitogen-activated protein kinase (MAPK) pathways and activation of MAPK by dual phosphorylation can enhance the transcriptional activity of activator protein (AP)-1 [4]. The transcription factor, c-Fos/AP-1, directly controls the expression of inflammatory cytokines, such as TNF-α, interleukin (IL)-1β and IL-6, by binding directly to AP-1 motifs in the promoter region of these genes. Aikawa *et al*[5]. designed and synthesized a selective inhibitor of c-Fos/AP-1, termed T-5224, using three-dimensional (3D) pharmacophore modeling based on a crystal structure of the AP-1–DNA complex, and found that selective inhibition of c-Fos/AP-1 resolved disease in a mouse model of arthritis. Inhibition of matrix-degrading matrix metalloproteinases (MMPs) is essential for effective disease-modifying anti-rheumatic drugs. T-5224 inhibited the transactivation of matrix-degrading MMPs via a promoter AP-1 binding motif and protected the joints in a mouse model of rheumatoid arthritis. Moreover, T-5224 decreased levels of TNF-α and efficiently protected mice from arthritic joint destruction.

In the present study, we investigated whether T-5224 has an inhibitory effect on LPS-induced AKI in mice by suppressing the inflammatory response, including TNF-α and other downstream effectors. Furthermore, as LPS administration can trigger an influx of neutrophils into organs such as the kidney, a process directed by the local expression of adhesion molecules on endothelium, we tested the effect of T-5224 on intercellular adhesion molecule (ICAM)-1 expression in the injured kidney.

## Methods

### Animals

This study was approved by the Institutional Animal Care and Use Committee and performed according to the Kobe University Animal Experimental Regulation (Kobe, Japan). Male C57BL/6 mice (CLEA Japan, Inc., Tokyo, Japan) aged 7 weeks were used throughout the study. They were maintained on standard rodent chow and allowed free access to water.

### Reagents

LPS (*Escherichia coli* 0111:B4) was purchased from Sigma-Aldrich Co. (St. Louis, MO,USA).T-5224, 3-{5-[4-(Cyclopentyloxy)-2-hydroxybenzoyl]-2-[(3-hydroxy-1,2-benzisoxazol-6-yl) methoxy]phenyl} propionic acid, was synthesized and kindly supplied by Toyama Chemical Co., Ltd (Toyama, Japan) [5]. T-5224 is insoluble in water; therefore this reagent was dissolved in a polyvinylpyrrolidone solution, and adjusted to a concentration of 30 mg/ml.

### Animal protocols, blood chemistry and measurement of serum cytokine levels

First we examined the peak of serum TNF-α levels after intraperitoneal administration a 6 mg/kg dose of LPS (1.25 mg/ml) [6] using a commercially available enzyme-linked immunosorbent assay (ELISA) kit (R&D systems, Inc., Minneapolis, MN, USA). For the preparation of serum, mice were anesthetized with an intraperitoneal injection of sodium pentobarbital (Dainippon Sumitomo Pharma Co., Ltd., Osaka, Japan) and blood samples were collected from mice via the femoral artery. One-milliliter aliquots of blood samples were centrifuged (6,700 × *g* for 3 min) and then supernatants were stored as serum at −80°C until used for analysis.

To evaluate the effect of T-5224 on LPS-induced AKI, mice were assigned to one of three groups (control group, LPS group and T-5224 group). Mice in LPS group were administered orally with polyvinylpyrrolidone solution in the same volume of T-5224 solution immediately after LPS injection, while in the T-5224 group mice were administered orally with T-5224 (300 mg/kg) in the same manner. In the control group, mice received polyvinylpyrrolidone solution orally soon after intraperitoneal saline injection. Blood samples were collected for each measurement at the optimal time. Serum samples from all groups were sent to a commercial laboratory service (Special Reference Laboratories, Tokyo, Japan) to measure serum blood urea nitrogen (BUN) levels. Serum creatinine (Cr) concentrations were also examined by enzymatic method in their laboratory. Serum IL-1β, IL-6 and IL-10 were measured using ELISA. ELISA kits were purchased from R&D Systems, Inc. for IL-1β and IL-6 measurements and Life Technologies (Grand Island, NY, USA) for IL-10 and were used according to the manufacturer's protocols.

### Real-time PCR for ICAM-1 expression

Quantitative real-time PCR was performed to examine ICAM-1 mRNA expression in LPS-induced AKI. The kidney was removed at 6 h after LPS or saline injection [6]. Total RNA from kidney was isolated using Trizol reagent (Life Technologies), according to the manufacturer's protocol. First strand cDNA was synthesized from 5 μg total RNA, using random hexamers with PrimeScript (Takara Bio Inc., Otsu, Japan). Real-time RT-PCR was performed on StepOnePlus (Life Technologies) using TaqMan Universal PCR Master Mix (Life Technologies) and TaqMan gene expression assays (Life Technologies) for ICAM-1 (assay ID: Mm00516023_m1) and β-actin (assay ID: Mm00607939_s1) were used for quantification of mRNA expression of the respective genes, according to the manufacturer's protocol. ICAM-1 levels were normalized by those of β-actin. Data were shown as the quantity relative to the control group.

### Histology

For histopathological observation, the mice were sacrificed and kidneys were removed at 48 h after LPS injection. Each tissue was fixed in 10% formalin, embedded in paraffin, sectioned, and then stained with hematoxylin and eosin for morphological examination.

### Statistical analysis

All data were expressed as mean ± standard deviation (SD). The Kruskal–Wallis test was used to test for overall group differences and the Steel-Dwass test was used to test for between-group differences. Values of *P*<0.05 were considered to represent statistical significance.

## Results

In this study, no mice in LPS group and T-5224 group died at 48 h after intraperitoneal administration of LPS with 6 mg/kg.

### T-5224 dose-dependently inhibits LPS-induced serum TNF-α

**Figure 1:**
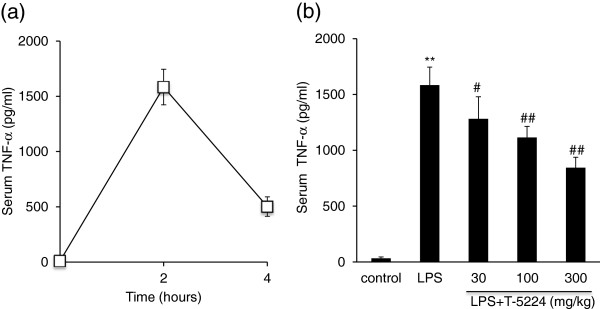
**T-5224 attenuates LPS-induced TNF-α. a,** determination of the point for TNF-α measurement. **b,** dose-dependent effects of T-5224 on TNF-α at 2 h after intraperitoneal LPS injection. Serum TNF-α levels peaked after LPS injection at 2 h (1583.8 ± 161.5 pg/ml). T-5224 attenuated LPS-induced TNF-α release dose-dependently (n = 7–8 per group). **P < 0.01 vs. control group. #*P* < 0.05 vs. LPS group. ##*P* < 0.01 vs. LPS group.

Serum TNF-α levels in mice peaked at 2 h after LPS injection (Figure 1a). Therefore, we examined the serum TNF-α levels at this point in all further experiments. Next, we investigated the dose-dependent effects of T-5224 on TNF-α expression in endotoxin-induced AKI. All doses of T-5224 significantly inhibited serum TNF-α levels compared to controls (Figure 1b). We also confirmed that T-5224 inhibited TNF-α dose dependently, as a dose of 300 mg/kg was more effective in reducing TNF-α production than that of 30 or 100 mg/kg (Figure 1b).

### T-5224 reduced serum BUN and Cr and pro-inflammatory cytokines

**Figure 2:**
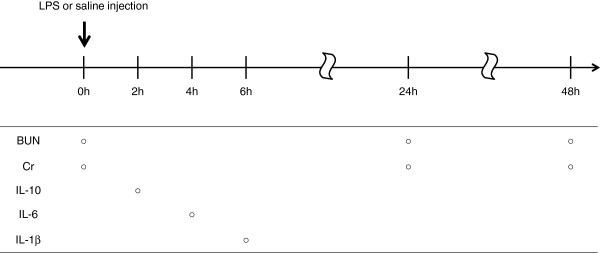
**Time course for blood collection.** LPS or saline was injected to mice at 0 h. Subsequently, blood was taken at different time points for the measurement of BUN, Cr, IL-10, IL-6 and IL-1β.

**Figure 3:**
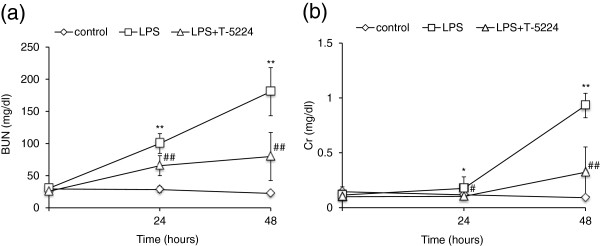
**Effect of T-5224 on serum BUN and Cr levels after LPS injection.** LPS induced serum BUN and Cr levels. **a,** BUN in the T-5224 group was significantly lower than that in the LPS group at both 24 h and 48 h (24 h, 65.5 ± 13.8 mg/dl vs. 100 ± 15.5 mg/dl; 48 h, 79.8 ± 41.9 mg/dl vs. 180.7 ± 37.4 mg/dl). **b,** T-5224 also attenuated the increase of serum Cr induced in LPS group (24 h, 0.13 ± 0.04 mg/dl vs. 0.21 ± 0.10 mg/dl; 48 h, 0.32 ± 0.23 mg/dl vs. 0.93 ± 0.11 mg/dl) (n = 6–12 per group). *P < 0.05 vs. control group. **P < 0.01 vs. control group. #*P* < 0.05 vs. LPS group. ##*P* < 0.01 vs. LPS group.

**Figure 4:**
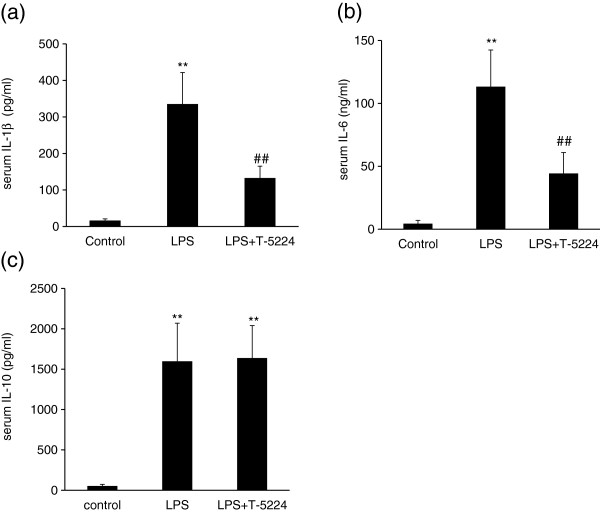
**Effect of T-5224 on serum cytokines following LPS administration.a,** the administration of T-5224 decreased serum IL-1β (132.7 ± 32.3 pg/ml vs. 335.5 ± 85.9 pg/ml). **b,** IL-6 levels were decreased after T-5224 treatment (44.4 ± 16.6 ng/ml vs. 113.4 ± 29.1 ng/ml) as compared with the LPS group. **c,** serum IL-10 levels in the T-5224 group were similar to the LPS group (1637.7 ± 402.3 pg/ml vs. 1597.7 ± 471.8 pg/ml) (n = 6–9 per group). **P < 0.01 vs. control group. ##*P* < 0.01 vs. LPS group.

Blood samples were collected for the measurement of serum BUN, Cr, IL-1β, IL-6 and IL-10 at the various optimal times (Figure 2). We examined serum BUN and Cr levels to evaluate kidney function. At the time of LPS or saline injection, serum BUN and Cr levels were comparable in all groups (Figure 3). In the LPS group, however, serum BUN and Cr levels were significantly increased at 24 h after LPS injection and further increased at 48 h, signifying kidney damage, while in the control group serum BUN and Cr levels remained low. The administration of T-5224 reduced serum BUN and Cr levels at both 24 h and 48 h. For a better understanding of potential mechanisms whereby T-5224 exerted a protective effect on renal function, we also analyzed the levels of serum TNF-α and other downstream cytokines. Serum TNF-α levels in the LPS group were significantly higher than in the control group, while treatment with T-5224 significantly attenuated its expression dose-dependently (Figure 1b). LPS also induced a significant increase in serum IL-1β and IL-6 levels compared with the control group. In contrast, T-5224 treatment inhibited LPS-induced increases of these cytokines (Figure 4a, b).

We also examined the expression of IL-10, an anti-inflammatory cytokine, and found that stimulation with LPS resulted in an equally significant up-regulation of IL-10 in the LPS and T-5224 groups, compared with the control group.

### T-5224 inhibits ICAM-1 mRNA in endothelium

**Figure 5:**
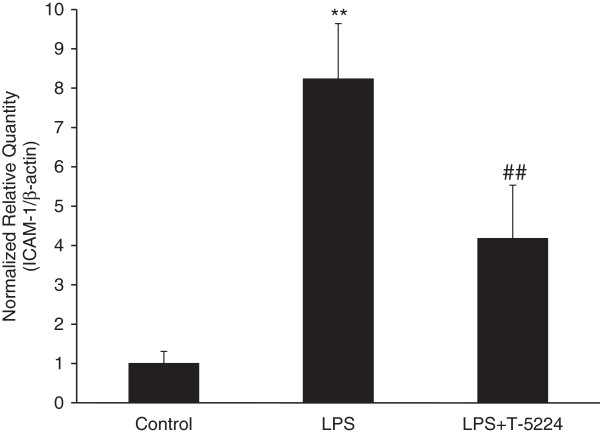
**Effect of T-5224 on ICAM-1 mRNA expression in injured kidney.** Bars represent the relative ratio of ICAM-1 mRNA to the control group. T-5224 significantly inhibited LPS-induced ICAM-1 mRNA expression in the kidney (4.2 ± 1.4 vs. 8.2 ± 1.4) (n = 4–6 per group). **P < 0.01 vs. control group. ##*P* < 0.01 vs. LPS group.

To determine whether T-5224 was capable of inhibiting the infiltration of neutrophils into the injured kidney, we measured mRNA expression of ICAM-1 by quantitative real-time RT-PCR (Figure 5). Following LPS challenge, ICAM-1 mRNA levels in the kidney were significantly higher than in the control group. However, T-5224 significantly inhibited LPS-induced ICAM-1 mRNA expression.

### T-5224 attenuated LPS-induced histopathological changes in kidney

**Figure 6:**
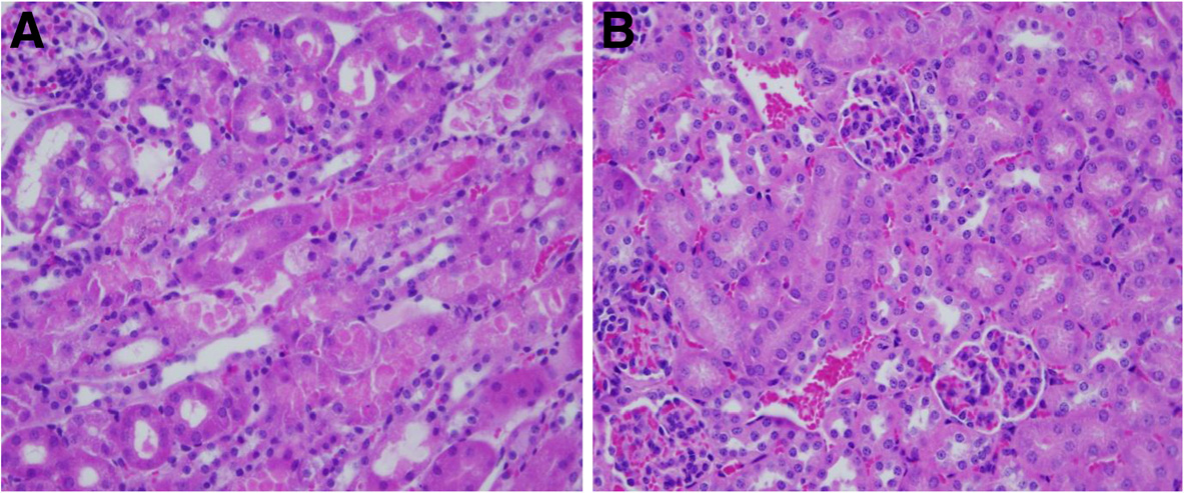
**Renal injury after LPS injection.A,** LPS caused tubular injury as manifested by tubular degeneration and dilatation. **B,** In contrast, there was less tubular damage in T-5224 group. The sections shown were harvested 48 h after LPS injection and stained with H&E. Magnification: ×400.

To evaluate histopathological characteristics of kidney in mice, the tissue sections stained with hematoxylin and eosin were made (Figure 6). After LPS injection, histopathological examination revealed kidney damage characterized by renal tubular degeneration and dilatation with variable flattening of the cytoplasm and loss of brush border. In contrast, treatment with T-5224 reduced the extent of kidney injury.

## Discussion

In the present study, we demonstrated that a selective c-Fos/AP-1 inhibitor, T-5224, suppressed LPS-induced TNF-α production dose-dependently. The inhibition of TNF-α is considered an effective treatment for septic AKI. The current study indicates that renal protection induced by T-5224 administration during endotoxemia occurs by controlling the expression of TNF-α and other downstream mediators.

Sepsis has been identified as the most common cause of AKI in intensive care units. Moreover, the combination of sepsis and AKI is associated with a very high mortality rate [1]. There is, therefore, an urgent need to identify novel therapeutic interventions with the potential to attenuate septic AKI. Numerous cytokines released from leukocytes and renal tubular cells in the injured kidney function as essential components of the initiation and extension of inflammation in AKI [7]. Hence, the pharmacological inhibition of these inflammatory mediators is a potential therapeutic target in the treatment of endotoxemia with bacterial infection. Recent studies indicated TNF-α is critical in mediating LPS-induced kidney injury [8]. Inhibition of TNF-α by genetic or antibody techniques inhibited renal injury following ischemia [9], cisplatin [10] or LPS [11] administration.

Aikawa *et al*. designed and synthesized a selective c-Fos/AP-1 inhibitor termed T-5224 using 3D pharmacophore modeling based on the crystal structure of the AP-1–DNA complex [5]. c-Fos/AP-1 controls the expression of inflammatory cytokines including TNF-α by binding directly to AP-1 motifs in the promoter of the gene, and T-5224 treatment resolved arthritis in a mouse model. The transcription factor AP-1 is also activated by LPS, leading to enhanced TNF-α transcription [12]. T-5224 inhibited the expression of TNF-α and other cytokines in a mouse arthritis model [5], suggesting it may also inhibit LPS-induced TNF-α production. Indeed, we demonstrated that T-5224 attenuated serum TNF-α levels dose-dependently following LPS administration and reduced serum BUN and Cr levels, indicating T-5224 can protect against LPS-induced AKI.

Apart from the inhibition of serum TNF-α during endotoxemia, T-5224 decreased other serum cytokines, namely IL-1β and IL-6. IL-1β is important in multiple organ failure and death during endotoxemia. IL-1β, a pro-inflammatory cytokine, evokes similar pathophysiological responses to TNF-α. Inhibition of IL-1β signaling by treatment with recombinant IL-1β receptor antagonist, resulted in decreased mortality in an animal model of endotoxic shock [13]. On the other hand, elevated plasma concentrations of IL-6 predict AKI in patients with severe sepsis [14] and mortality in sepsis correlates with the degree of IL-6 up regulation [15]. T-5224 showed a remarkable inhibition of IL-1β and IL-6 production in mice challenged with LPS, suggesting that T-5224 can protect renal function against LPS, at least in part, via inhibiting both IL-1β and IL-6 production.

IL-10, an anti-inflammatory cytokine, plays an important role in improving survival in animals challenged with LPS and inhibits several macrophage functions including TNF-α production *in vivo* and *in vitro*[16]. However, one study has reported that TNF-α signaling can promote IL-10 production [17]. Considering this, there was a possibility that T-5224 might inhibit IL-10 production. However, T-5224 did not suppress IL-10 production in our study. This observation is of interest since modulating the balance between pro-inflammatory and anti-inflammatory responses and restoring immune homeostasis of patients is important in the treatment of sepsis.

The effect of T-5224 on ICAM-1 mRNA expression in the kidney was also examined. ICAM-1 is a member of the immunoglobulin-like supergene family of adhesion molecules known to mediate adherence of polymorphonuclear neutrophils (PMNs) to endothelial cells [18]. Both LPS and TNF-α increase the cell surface expression of ICAM-1 [19], which is one of several downstream effectors induced by TNF-α. The present study demonstrated that mRNA expression of ICAM-1 was significantly increased in the kidney at 6 h after LPS injection and was markedly attenuated by treatment of T-5224. The role of ICAM-1 in PMN accumulation has been suggested in endotoxemic AKI and a deficiency of ICAM-1 may confer protection against LPS-induced AKI, by decreasing neutrophil adhesion and abrogating signaling events that occur in endothelium upon ligation of ICAM-1. Thus, our results suggested that T-5224 might also protect mice against LPS-induced AKI by decreasing ICAM-1 expression.

We also examined renal histological change at 48 h after LPS injection. As previous study reported, LPS induced severe renal tubular injury [20]. In this study, mice in LPS group also developed tubular injury. However, less tubular damage was observed in T-5224 group. Furthermore, pathological findings were well correlated with reduced expressions of TNF-α and other downstream effectors. These results indicated that T-5224 conferred protection against structural injury caused by LPS. Interestingly we observed few neutrophils in kidney, which suggests that ICAM-1 itself may cause renal injury through other as yet unknown neutrophil-independent mechanisms.

In the present study, T-5224 did not completely suppress all inflammatory mediators. One potential explanation for this could be that genes encoding TNF-α are also transcriptionally activated through nuclear factor (NF)-κB activation [21]. NF-κB, a transcription factor, is critically involved in the regulation of monocytic production of pro-inflammatory cytokines, such as TNF-α and IL-1β. T-5224 specifically inhibits the DNA binding activity of c-Fos/c-Jun, without affecting that of NF-κB/p65 [5]. There are also several limitations in our study. First, we only examined BUN and Cr for kidney function, although these markers are insensitive and non-specific for acute changes to kidney function. Therefore, these biomarkers may not detect an ongoing injury to the kidney. Second, T-5224 was administrated immediately after LPS injection. Considering the clinical situation, the delayed administration of T-5224 should also be investigated in the future. Third, intravenous administration is generally more effective than oral administration. However, we demonstrated that oral treatment with T-5224 was effective enough to improve kidney injury in the acute phase of sepsis. The development of T-5224 for intravenous drug use is now underway.

## Conclusions

We have shown that T-5224 attenuated LPS-induced AKI in mice. This protection was associated with reduced expressions of serum TNF-α and other downstream cytokines including IL-1β and IL-6. In addition, the anti-inflammatory cytokine IL-10 was not affected by T-5224. Moreover, renal ICAM-1 mRNA induced by LPS was suppressed by T-5224.

## Abbreviations

AKI: Acute kidney injury; AP-1: Activator protein-1; BUN: Blood urea nitrogen; Cr: Creatinine; ELISA: Enzyme-linked immunosorbent assay; ICAM-1: Intercellular adhesion molecule-1; IL: Interleukin; LPS: Lipopolysaccharide; MAPK: Mitogen-activated protein kinase; MMPs: Matrix-degrading matrix metalloproteinases; NF-κB: Nuclear factor-κB; PMN: Polymorphonuclear neutrophil; 3D: Three-dimensional; TNF-α: Tumor necrosis factor-α, H&E, hematoxylin and eosin.

## Competing interests

The authors declare that they have no competing interests (SS holds a patent relating to the content of the manuscript, but has never received reimbursements, fees, funding, or salary from a company relating to the content of the manuscript).

## Authors' contributions

HM, JM and UM designed and coordinated the study. HM was responsible for blood sample collection, and data acquisition. JM and UM were involved in the interpretation of the data and manuscript drafting. JM, UM, KN and NM reviewed the manuscript. SS conceived the study. All authors read and approved the final manuscript.

## Pre-publication history

The pre-publication history for this paper can be accessed here:

http://www.biomedcentral.com/1471-2369/13/153/prepub
